# Oncolytic Viruses for Cancer Therapy in Dogs

**DOI:** 10.3390/v18050518

**Published:** 2026-04-30

**Authors:** Daria O. Neymysheva, Galina V. Ilyinskaya, Viktoria A. Sarkisova, Elena A. Mukhina, Sofia A. Romanen-kova, Peter M. Chumakov

**Affiliations:** 1 Engelhardt Institute of Molecular Biology, Russian Academy of Sciences, Moscow 119991, Russia; neymyshevadaria@gmail.com (D.O.N.); ilyinskaya@yahoo.com (G.V.I.); alice-lyddell@yandex.ru (V.A.S.); 2 Center for Precision Genetic Technologies for Medicine, Engelhardt Institute of Molecular Biology, Russian Academy of Sciences, Moscow 119991, Russia; 3 Faculty of Biology, Lomonosov Moscow State University, Moscow 119234, Russia; 4 Institute of Immunology Federal Medical-Biological Agency of Russia, Moscow 115522, Russia; kaysova@yandex.ru; 5 Department of Pharmacology, A.P. Nelyubin Institute of Pharmacy, I.M. Sechenov First Moscow State Medical University (Sechenov University), Moscow 119048, Russia; sophiaromanenkova@gmail.com

**Keywords:** oncolytic virus, canine cancer, virotherapy, immunotherapy, veterinary oncology

## Abstract

Cancer remains the leading cause of death in domestic dogs. Conventional therapeutic approaches, including surgery, chemotherapy, and radiotherapy, frequently fail to achieve sustained remission or stabilization. Oncolytic virotherapy, a rapidly advancing therapeutic modality in human oncology, is emerging as a novel strategy in veterinary medicine. This systematic review summarizes current knowledge on the application of oncolytic viruses (OVs) in canine cancer treatment, focusing on their mechanisms of action, safety profiles, and clinical efficacy. We evaluate diverse OV platforms, including myxoma virus, reovirus, vesicular stomatitis virus, canine adenoviruses, vaccinia virus, Sendai virus, and Newcastle disease virus, across preclinical and clinical studies in dogs with various malignancies. While several OVs have demonstrated favorable tolerability and modest antitumor activity, key challenges such as pre-existing immunity, optimization of dosing regimens, and rational combination strategies remain to be addressed. This review emphasizes the translational significance of canine studies for both veterinary and human oncology, underscoring the critical need for rigorously designed clinical trials to refine virotherapy protocols and expand therapeutic options for canine cancer patients.

## 1. Introduction

Cancer is a leading cause of death in domestic dogs, and approximately one in four dogs will develop cancer during their lifetime [1]. Despite the high prevalence of canine cancer, existing treatment protocols, including chemotherapy, radiation therapy, and surgery, frequently fail to achieve stable remission or even disease stabilization.

The limited efficacy of conventional treatments stems from several factors that include intrinsic or acquired tumor resistance to therapeutic interventions, presence of multifocal lesions, extensive invasion of surrounding tissues, and anatomical inaccessibility for surgical or radiotherapeutic intervention [2]. Conservative treatment strategies often result in progressive disease and declining quality of life, while surgical resection carries the inherent risk of promoting metastatic dissemination [3].

There is a compelling need for innovative and efficacious anti-cancer treatments in veterinary medicine. Notably, research and development in this field remain substantially less advanced than in human oncology. Historically, standard veterinary treatment protocols have been adapted from human cancer therapy. However, novel therapeutic paradigms, including immunotherapy and targeted molecular therapy, which have been actively pursued and successfully implemented in human oncology, remain in early developmental stages in veterinary practice. Commercially available immunotherapeutic agents, such as monoclonal antibodies, targeted inhibitors, and checkpoint inhibitors, are substantially limited compared to the extensive therapeutic armamentarium available to human patients [4].

Oncolytic virotherapy represents an innovative therapeutic approach to canine cancer that leverages oncolytic viruses (OVs), evolutionarily diverse viral agents capable of selectively inducing malignant cell death while sparing normal cellular populations [5]. OVs encompass representatives from multiple viral families, including Adenoviridae, Poxviridae, Picornaviridae, Paramyxoviridae, and others. The safety and efficacy of several OVs have been established in human clinical trials, and certain oncolytic viral therapeutics have received regulatory approval in the United States, Latvia, China, and Japan [6,7,8,9]. Additional OVs are currently undergoing active investigation in various human clinical trials [10].

The extensive translational research and development of diverse OVs for human cancer treatment has catalyzed investigation into their potential application in veterinary oncology. This review aims to synthesize existing clinical experience with virotherapy in dogs and evaluate the prospects for integrating oncolytic viruses into veterinary cancer treatment protocols.

## 2. General Mechanisms of Virus-Mediated Oncolysis

**Figure 1:**
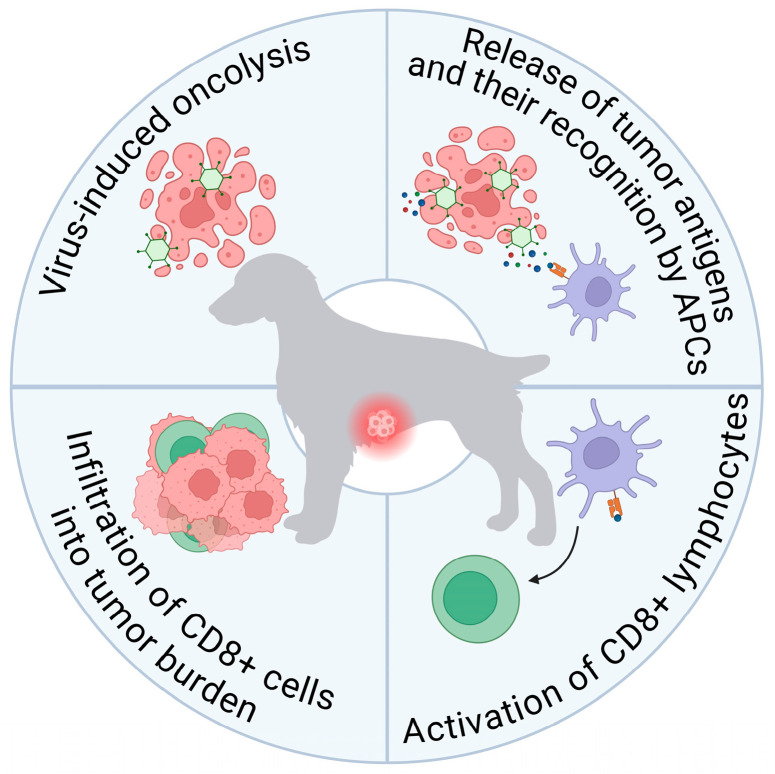
Mechanisms of viral-induced oncolysis.

Oncolysis is mediated by multiple complementary mechanisms, including direct malignant cell lysis and the activation of antitumor immune responses [11] (Figure 1). Direct lysis occurs because certain malignant cells, as a consequence of malignant transformation, often express elevated levels of specific viral receptors (such as CD155, CD46, and others) that are essential for viral infection establishment. Additionally, many tumor cells exhibit impaired intrinsic antiviral defenses, predominantly due to the disruption of type I interferon (IFN) signaling pathways. These deficiencies permit efficient viral replication within tumor cells while restricting replication in normal cells with intact antiviral mechanisms [12].

Beyond direct oncolysis, OVs activate diverse immune response pathways, making immune stimulation an equally critical mechanism of antitumor activity [13]. The viral destruction of tumor cells results in the release of tumor-associated antigens into the microenvironment. When immune effector cells are present, these antigens are presented to antigen-presenting cells, triggering antitumor immune responses. Consequently, the immune system becomes activated not only against viral antigens but also against released tumor antigens, generating a more robust antitumor response [14].

Virally infected tumors recruit substantial immune infiltration, transforming an immunologically suppressive (“cold”) microenvironment into an immunologically active (“hot”) one, thereby enhancing antitumor immune responses. Viral infection can reprogram tumor-associated immune populations, for example, shifting tumor-associated macrophages from the immunosuppressive M2 phenotype to the pro-inflammatory M1 phenotype, thereby reducing immune evasion mechanisms [14,15]. This multifaceted mechanism of action renders OVs uniquely suited as anticancer agents, as they simultaneously disrupt multiple critical aspects of tumor biology and immune regulation.

## 3. Clinical Applications of OVs in Dogs

The field of oncolytic virotherapy has primarily focused on developing innovative approaches for cancer treatment in humans. However, because of host-range limitations, not all OVs designed for human use are suitable for therapy in dogs. Conversely, some OVs are being developed specifically for canine patients and are not appropriate for use in humans. Viruses that can be used in both species are particularly valuable, as they make the dog a relevant preclinical model for evaluating viral safety and antitumor efficacy. Table 1 summarizes the OVs that have so far been considered for cancer treatment in dogs, and in the following sections we discuss these viral platforms in more detail with a focus on their application in canine oncology.

### 3.1. Myxoma Virus

The myxoma virus (MYXV) is a large, enveloped, double-stranded DNA virus belonging to the Poxviridae family. The natural host is Brazilian cottontail rabbit (*Sylvilagus brasiliensis*), in which the virus causes no pathological manifestations. However, in European and domestic rabbits, MYXV causes myxomatosis, a frequently lethal infection [16]. Clinical manifestations include nodular skin lesions, facial and anogenital edema, conjunctivitis, ocular discharge, fever, and anorexia, typically progressing to death.

The high lethality of myxomatosis results from MYXV encoding an extensive repertoire of immunomodulatory proteins that disrupt innate immunity and suppress apoptotic pathways. MYXV principally impairs innate immune responses through molecular mimicry of crucial immunoregulatory cytokines, including TNFα and IFN-γ [40]. The virus attaches to cell surfaces through glycosaminoglycan-mediated interactions, binding heparin and chondroitin sulfate moieties [41].

Despite being highly pathogenic to rabbits, MYXV exhibits potent oncolytic properties against diverse human malignancies, both in vitro and in vivo [17,42].

Amy MacNeill et al. investigated the safety and toxicity of MYXV with *SERP2* deletion in ten dogs with soft tissue sarcomas [17,43]. The study included ten dogs, which were divided into two cohorts: five dogs received intratumoral injection, and five dogs received postoperative viral treatment at the surgery site due to anatomical constraints that prevented wide excision of the tumor.

The researchers employed a recombinant MYXVΔserp2 strain, which demonstrated more pronounced cytopathic effects against canine malignant cells compared to wild-type virus [44]. The *SERP2* gene encodes a protein that inhibits interleukin-converting enzymes (ICE), which are essential for processing pro-interleukin-1 to mature interleukin-1. This IL-1 pathway disruption is crucial for myxomatosis progression and directly impacts immune surveillance in rabbits [45]. Deletion of *SERP2* gene was designed to attenuate potential serious complications during virotherapy.

Overall, treatment with MYXVΔserp2 remained safe, with seven dogs experiencing grade 2 AEs of some kind and three dogs having grade 1 AEs or no detectable AEs at all. Although the evaluation of efficacy was not the primary goal of the study, the authors reported changes in the tumor diameter of patients who received intratumoral viral injections. However, changes in tumor diameter among most patients were insignificant; only one dog showed a mark decrease in tumor size (2.7 cm). The histopathological examination of tumor biopsies revealed only modest infiltration by inflammatory immune cells at later timepoints. Anti-MYXV antibodies were detected in two dogs from the postoperative treatment group. No significant alterations in blood leukocyte populations were observed in any cohort. Viral DNA was detected at low levels in peripheral blood relative to controls. These findings suggest that the efficacy of MYXVΔserp2 against canine soft tissue sarcomas, achieved through localized administration, was limited. The modest antitumor effect may be attributable to innate immune-mediated virus inactivation prior to adaptive immune response initiation. However, combination therapy pairing MYXVΔserp2 with immune checkpoint inhibitors may potentially enhance efficacy, based on evidence from canine malignant cell lines [46].

### 3.2. Vaccinia Virus

The vaccinia virus (VACV), an enveloped double-stranded DNA virus of the Poxviridae family, has an extensive history of use as a smallpox vaccine [27,47]. Recently, VACV has emerged as a leading oncolytic therapeutic agent, with multiple recombinant variants currently under clinical trials [27,47,48].

Two well-characterized vaccine strains predominate: the Western Reserve (WR) strain and the more virulent Lister strain, both historically employed for smallpox eradication. While VACVs specific cellular receptor is poorly characterized, the virus utilizes glycosaminoglycans (such as heparin sulfate and chondroitin sulfate) for cell surface attachment, similar to MYXV. These ubiquitous extracellular matrix molecules expand viral tropism across diverse cell types, including malignant cells. Certain VACV strains additionally enter cells via glycopeptide laminin or macrophage receptor with collagenous structure (MARCO) [49,50].

The recombinant vaccinia virus TG6002 has undergone both in vitro and in vivo evaluation on canine cancer models to assess safety and efficacy [51,52]. TG6002 was engineered with targeted deletions in *J2R*, *I4L*, and *F4L* genes. Inactivation of *J2R* (encoding viral thymidine kinase) reduces VACV’s toxicity, while enhancing its tumor tropism. The *I4L* and *F4L* genes encode ribonucleotide reductase-like proteins, modifications that further increase tumor selectivity. To enhance therapeutic potential, the FCU1 gene was inserted into the TG6002 genome, encoding a bifunctional protein with deaminase and uracil phosphoribosyl transferase activities. This enzyme converts the non-toxic prodrug 5-fluorocytosine (5-FC) into active chemotherapeutic agents, 5-fluorouracil (5-FU) and 5-FU monophosphate, which inhibit nucleic acid synthesis and enhance viral antitumor efficacy [53].

Safety profiling of TG6002 in four healthy beagle dogs demonstrated no significant adverse effects [54]. To date, a single clinical efficacy trial with recombinant VACV (TG6002) has been conducted in dogs with malignant solid tumors [51]. Thirteen dogs with various solid tumors previously treated with other modalities received TG6002 containing 5-FC delivered via intratumoral infection. Dogs were stratified into three dosing groups: Group 1 received two or three intratumoral injections of 5 × 10^6^ PFU/kg; Group 2 received a single injection of 5 × 10^7^ PFU/kg; Group 3 received three weekly injections of 5 × 10^7^ PFU/kg. Median survival across all groups was 115 days.

Viral replication was confirmed in six dogs via the detection of the viral genome in peripheral blood at day 3, with dose-dependent increases in viral load. Intratumoral 5-FU concentration increased in the higher-dose cohort; however, no 5-FU-related systemic toxicity was observed, and 5-FU was not detected in peripheral blood. Histological evaluation demonstrated tumor necrosis in nine cases.

TG6002 administration proved well-tolerated, with no fatal adverse events. Grade 4 adverse events occurred in 3.3% of patients. Notably, 5-FU production was restricted to the tumor microenvironment, substantially reducing the risk of systemic toxicity. While the treatment was generally safe, its clinical antitumor efficacy was modest: following treatment, patients exhibited partial response (one case in Group 3), stable disease, or progressive disease.

### 3.3. Reoviruses

Reoviruses are small, non-enveloped, segmented, double-stranded RNA viruses that commonly infect humans, frequently without symptomatic manifestations [55]. Reovirus infection is prevalent across diverse mammalian species, including canines [18,56].

Reovirus attaches to target cells through interaction between the viral attachment protein σ1 and junctional adhesion molecule A (JAM-A) [57,58]. JAM-A is a transmembrane protein belonging to the immunoglobulin superfamily that localizes in the tight junctions of endothelial and epithelial cells [59,60]. Ubiquitous sialic acids serve as co-attachment factors, binding viral capsids to stabilize virus–cell interactions prior to receptor engagement [61].

During the 2000s, researchers identified the oncolytic activity of reovirus serotype 3 (Dearing strain), commercially designated Reolysin [18,62]. Hwang and colleagues demonstrated the oncolytic potential of the Dearing strain against diverse canine tumor types, including mastocytomas [63], mammary gland neoplasms [64], canine lymphomas [65], and solid tumors [66] in in vitro models.

In subsequent in vivo studies, these researchers evaluated virotherapy safety using Reolysin in nineteen dogs of various breeds with diverse tumor types [67]. Depending on tumor location, dogs received either intravenous infusion or intratumoral injection. Viral biodistribution was quantified by reverse transcription polymerase chain reaction (RT-PCR) analysis of saliva, serum, feces, and urine. Viral RNA was detected in nearly all serum samples. Depending on tumor burden and clinical status, dogs received either single or multiple virus administrations during the observation period. Serum neutralizing antibody assays revealed elevated anti-reovirus antibody titers in almost all dogs, potentially impairing viral trafficking to tumor sites. Notably, several dogs possessed pre-existing anti-reovirus antibodies prior to treatment, which could impact therapeutic efficacy.

Six dogs demonstrated an overall improvement and reduction in cancer-related symptoms. Tumor size reduction was observed in five dogs with various solid tumors. Treatment was individualized based on disease and patient wellbeing. Dogs experienced mild adverse effects during treatment, including fever, vomiting, diarrhea, tumor hemorrhage, and injection site discomfort when intravenously administered; however, treatment was generally well-tolerated. Reolysin demonstrated oncolytic activity against a wide range of canine solid tumors that are otherwise refractory to conventional therapy. The observed tumor size reduction and improved quality of life suggest efficacy against different canine malignancies, warranting further investigation in additional tumor types.

Notably, combination therapy pairing Reolysin with drugs that enhance reovirus cytotoxicity represents a promising strategy. For example, the exposure of diverse canine melanoma cell lines to Reolysin combined with the ATM inhibitor KU60019 produced significantly greater reductions in cell proliferation compared to single-agent treatment [67,68]. However, the further in vivo investigation of this combined approach is necessary to establish clinical utility.

### 3.4. Vesicular Stomatitis Virus

Vesicular Stomatitis Virus (VSV), a member of the Rhabdoviridae family, causes vesicular stomatitis in livestock, including horses, cattle and swine [21]. Two serotypes—Indiana and New Jersey— predominate. VSV exhibits high transmissibility among ungulate species, producing characteristic blisters in the oral cavity, and on the udder and hooves. By contrast, human VSV infections are typically asymptomatic or mild, with life-threatening complications being exceptionally rare [69].

Structurally, VSV contains a single-stranded negative-sense RNA genome within a bullet-shaped virion. VSV enters cells by attaching to low-density lipoprotein receptor (LDLR) and subsequently utilizing clathrin-mediated endocytosis, facilitated by dynamine-2 and actin [70]. VSV demonstrates preferential tropism for human malignant cells [71]. The enhanced susceptibility of cancer cells to VSV infection, combined with facile genetic manipulation, renders VSV an attractive oncolytic candidate.

One construct, recombinant VSV expressing human interferon β (IFNβ) and sodium-iodide transporter (NIS), has been evaluated in dogs for toxicity and antitumor activity. IFN-β expression by infected tumor cells provides a protective mechanism for neighboring normal cells by triggering IFN-mediated antiviral pathways. NIS expression enables the visualization of viral distribution via radioisotope tomography following radioactive iodine administration. Systemic administration of VSV-hIFN-β-NIS is currently under evaluation in human clinical trials [72].

The absence of naturally occurring VSV disease in dogs provides a rational basis for exploring VSV as a potentially safe anticancer agent in canine patients. The safety and pharmacokinetic properties of recombinant VSV-hIFNβ-NIS were evaluated in five purpose-bred beagle dogs [73]. Virus was administered intravenously using a tenfold dose escalation, ranging from 10^8^ to 10^11^ TCID_50_. One dog required euthanasia due to dose-limiting toxicity (severe diarrhea causing dehydration and shock). Dogs receiving doses of 10^10^ TCID_50_ or lower experienced no significant adverse events. During treatment, dogs exhibited fever, nausea, and vomiting. Mild scarified dermatitis and oral lesions were observed. No neurotoxic manifestations were detected. Viral RNA, indicative of viremia, became undetectable in blood after ten days; viral replication assays confirmed blood clearance by 24 h.

In a subsequent trial, the oncolytic efficacy of VSV-IFNβ-NIS expressing human (VSV-hIFNβ-NIS) or canine (VSV-cIFNβ-NIS) interferon was assessed in ten dogs with diverse malignancies, including anal adenocarcinoma, multiple myeloma, B-cell and T-cell lymphomas, metastatic osteosarcoma, and multifocal cutaneous melanoma [74]. Virus was administered intravenously at 1 × 10^10^ TCID_50_ per 0.5 m^2^.

During treatment, nine dogs experienced mild fever without significant complications. One dog exhibited tenfold elevation in alanine aminotransferase, indicating Grade 3 hepatotoxicity according to VCOG criteria [75]. Clinical outcomes varied: five dogs achieved disease stabilization, two dogs with T-cell lymphoma achieved rapid remission but subsequently relapsed, and three dogs with B-cell lymphoma demonstrated disease progression.

A subsequent clinical investigation of VSV-cIFNβ-NIS as neoadjuvant therapy was conducted in 28 dogs with osteosarcoma at various stages [76]. Dogs received a single intravenous dose containing 1 × 10^9^ TCID_50_/kg.

Treated dogs experienced mild fever, transient hepatotoxicity, and mild lymphopenia that resolved spontaneously. Efficacy was compared to two control cohorts: one comprising dogs from the University of Minnesota Veterinary Medical Center (UMN VMC), receiving standard surgical therapy and adjuvant carboplatin chemotherapy, and another consisting of 157 dogs receiving protocols designed by the National Cancer Institute’s Comparative Oncology Trial Consortium (NCI-COTC).

Neoadjuvant VSV-IFNβ-NIS did not increase survival relative to control groups, nor did it decrease survival. However, the VSV-treated cohort showed a higher proportion of long-term survivors (35%) compared to UMN VMC (26%) and NCI-COTC (25%) groups.

T-cell infiltration into the tumor microenvironment correlated with improved survival in both treated and untreated NCI-COTC cohorts, with this association being more pronounced in VSV-treated animals. While VSV demonstrated promise as a potentially safe therapeutic option for canine neoplasms, additional research is required to fully characterize its efficacy across tumor types and establish its optimal clinical role.

### 3.5. Adenoviruses

Adenoviruses are non-enveloped double-stranded DNA viruses causing diverse diseases. Adenoviruses are extensively investigated autonomous oncolytic agents and as vectors for therapeutic gene delivery to tumors [77]. The primary adenovirus receptor is coxsackievirus and adenovirus receptor protein (CAR). In humans, CAR expression localizes to hepatocytes, cardiomyocytes, pancreatic duct and acinar cells, and the renal epithelium [78]. Elevated CAR expression, relative to normal tissues, occurs in ovarian, breast, and non-small cell lung cancers, as well as prostate cancer and osteosarcoma [79,80,81,82,83,84,85]. CAR hyperexpression in these cancers can potentially be exploited, while adenoviruses serve as an effective vehicle for delivering genes that can improve oncolytic virus therapy responses [86].

Dogs, like many mammalian species, serve as natural hosts for diverse adenoviruses and express the primary receptor for adenovirus type 2—CAR [25]. Two adenovirus types infect canines: canine adenovirus type 1 (CAV-1) and canine adenovirus type 2 (CAV-2) [26,77]. CAV-1 primarily affects the gastrointestinal tract, causing infectious canine hepatitis, while CAV-2 infections manifest as mild respiratory disease.

In a 2006 safety study of CAV-2 as an oncolytic agent, six healthy mixed-breed dogs were vaccinated. Nearly all possessed pre-existing anti-CAV-2 antibodies [24]. Post-vaccination, anti-CAV-2 antibody titers increased substantially [87], reflecting the high immunogenicity properties of CAV-2, which could substantially influence virotherapy outcomes.

Two recombinant CAV-2 constructs have been evaluated in dogs: ICOCAV15 and ICOCAV-17 [87,88]. These viruses contain modified CAV-2 with RGD motifs enabling continuous viral replication. Additionally, ICOCAV17 encodes human PH20 hyaluronidase, which enhances intratumoral dissemination.

The oncolytic activity and safety of ICOCAV-15 and ICOCAV-17 were investigated in various clinical trials. ICOCAV15 was administered intratumorally to eight dogs with multiple carcinomas [89]. Twenty-five percent exhibited partial response, while the remaining cohort showed disease stabilization. Six dogs lived longer than nine months, with survival times exceeding median survival for similarly diagnosed dogs receiving chemotherapy. Tumor size reduction was noted in nasal and squamous cell carcinomas, accompanied by improved patient wellbeing.

In another investigation, ICOCAV17 was delivered using adipose-derived mesenchymal stem cells (MSCs) as carriers [90]. Treatment was administered as monotherapy in most cases; however, ten dogs concurrently received chemotherapy agents, including doxorubicin, tyrosine kinase inhibitors, cyclophosphamide, and prednisone. Twenty-seven dogs with various cancers received MSCs infected at a multiplicity of one viral particle per cell. MSCs containing ICOCAV17 were administered intravenously once weekly for four consecutive weeks.

Following treatment, 14.8% exhibited complete response, 11.1% showed partial response, 48.1% achieved disease stabilization, and 25.9% experienced disease progression. Nonetheless, significant clinical improvement was observed in the vast majority of patients from both groups.

Biopsy examination revealed tumor infiltration by MAC387-expressing immunocytes, indicating immunostimulatory treatment effects. Pre-treatment analysis identified neutralizing anti-CAV-2 antibodies. During therapy, these antibody titers increased. However, no clear correlation emerged between antibody presence and clinical response.

In another trial, ten French bulldogs with high-grade glioma received MCSs containing ICOCAV17 once weekly for eight weeks [47]. Clinical response was evaluated in seven dogs. Two dogs demonstrated disease progression, three achieved disease stabilization, and two exhibited partial response.

Similar to prior studies, treatment was well-tolerated with no severe adverse events. However, a correlation was noted between peripheral viral particle concentration and the pre-existing levels of anti-CAV2 antibodies. This correlation suggests that elevated baseline IgG titers of anti-CAV-2 antibodies are associated with reduced number of free viral particles and potentially decreased intratumoral viral load.

These findings suggest that CAV-2-based recombinant viruses have potential as oncolytic agents for diverse solid tumors. While elevated anti-CAV-2 antibody titers can impair virotherapy efficacy, additional investigation is required to elucidate this correlation. Investigation of CAV2-based therapy in lymphomas and mast cell tumors appears warranted.

### 3.6. Sendai Virus

Sendai virus, also designated as hemagglutinating virus of Japan (HVJ), is a paramyxovirus associated with respiratory infections in rodents, belonging to the Paramyxoviridae family of enveloped negative-sense single-stranded RNA viruses. Initially explored as a vaccine candidate against parainfluenza virus type 1 (HPIV-1) [30], current research focuses on its oncolytic properties, as Sendai virus remains non-pathogenic for humans [91].

Sialic acid and its protein derivatives represent primary Sendai receptors; these are more abundantly expressed on tumor cells than non-transformed cells [92]. Similar to other paramyxoviruses, Sendai requires specific proteases for the cleavage and activation of the F0 precursor into fusion proteins F1 and F2, which are necessary for viral cell entry [93]. Multiple serine proteases, including hepsin, matriptase, and TMPRSS, are overexpressed on diverse cancer cell surfaces, rendering malignant cells susceptible to Sendai infection [94].

Beyond direct oncolysis, Sendai-mediated tumor eradication principally relies on stimulating antitumor immune responses, activating cytotoxic T lymphocytes and natural killer cells. These properties have motivated clinical trials examining Sendai efficacy and safety in human patients with castration-resistant prostate cancer, chemotherapy-resistant malignant pleural mesothelioma, and melanoma [21,31,32].

Our research team conducted a study evaluating Sendai efficacy in treating six dogs with mast cell tumors [95]. The treatment protocol involved viral injection into the tumor mass, subcutaneously surrounding the tumor, or at the surgical resection site at a titer of 10^7^/mL. In five out of six cases, no disease recurrence occurred, and treatment was well-tolerated. However, one mixed-breed dog required euthanasia after eight weeks due to lymph node metastases, despite a modest reduction in metastatic nodule size during virotherapy. In several cases, multiple Sendai applications were necessary, resulting in disease stabilization and an improved quality of life.

To further investigate Sendai’s oncolytic potential in veterinary medicine, an assessment of its capacity to activate canine immune responses, its tolerability during systemic administration, and its efficacy against diverse solid and hematologic malignancies is essential.

### 3.7. Newcastle Disease Virus

Newcastle disease virus (NDV), another member of the Paramyxoviridae family, has been extensively studied for oncolytic properties for over more than fifty years. NDV is highly virulent among avian species, with pathogenic strains causing Newcastle disease with 100% mortality in infected birds [34]. NDV strains are classified by pathogenicity into two groups: Velogenic strains are maximally virulent with nearly 100% avian mortality; Mesogenic strains are moderately virulent but represent agricultural hazards. Lentogenic strains group are non-virulent or attenuated, and are primarily used for vaccination (including LaSota and Ulster strains) [36].

NDV host factors, similar to Sendai virus, include proteases and sialic acid derivates. Strain virulence correlates with amino acid sequence variations at proteolytic cleavage sites for F0 protein processing into F1 and F2 peptides. The specific protease cleavage site requirements differ among strains: Mesogenic and Velogenic strains utilize multiple proteases, while Lentogenic strains primarily employ trypsin-like proteases for cell entry.

NDV demonstrates efficacy and safety in human clinical trials for diverse malignancies, including colorectal cancer, glioblastoma, and melanoma [34,35]. Investigations of NDV oncolytic potential using canine cell lines has motivated subsequent in vivo studies. Multiple wild-type and recombinant NDV strains have undergone evaluation in three separate clinical studies in dogs with lymphoma, breast cancer, and intracranial neoplasms. During investigation of spontaneous canine breast cancer, avirulent NDV-MLS strain was administered intravenously and intratumorally once, followed by six days of clinical observation prior to surgical resection [96]. Viral administration of the virus caused no significant adverse effects or viral shedding. Biodistribution analysis revealed that the intravenous route ineffectively delivered virus to tumor lesions within 24 h. Following intratumoral injection, five of six treated dogs demonstrated varying degrees of tumor infiltration by lymphocytes, macrophages, neutrophils, and plasma cells, attributable to viral immune stimulation. After six days, NDV-specific antibodies were detected in only two dogs.

The oncolytic potential of recombinant Lentogenic NDV strain expressing urokinase plasminogen activator (rLAS-uPA) was evaluated in a two-stage clinical trial involving 20 dogs with diverse intracranial neoplasms [97]. The urokinase plasminogen activator system enhances viral dissemination and oncolytic potential. The study employed systematic intravenous dosing consisting of three administrations at 14-day intervals. The maximum tolerated dose was determined as 2 × 10^7^ PFU/mL. Following therapy, 17 dogs achieved disease stabilization, two showed partial response, and one demonstrated progressive disease. Rapid increases in antiviral antibody titers occurred following the first injection, persisting throughout both study phases. Six dogs died during the research period; post-mortem brain tissue examination by quantitative reverse transcription PCR (qRT-PCR) confirmed viral presence. In five out of six necropsy specimens, viral messenger RNA increased relative to GAPDH expression. Substantial elevations in cytokine concentrations, including TNF-α, IFN-α, and TRAIL/ApoL2, were detected in each cohort, potentially indicating antitumor immune response activation.

Each dog was monitored until death. In six instances, lifespan exceeded one year, with one case extending beyond two years. Notably, all received additional treatment, including surgery and, in some cases, radiation therapy.

## 4. Discussion

Oncolytic virotherapy represents a novel cancer immunotherapy approach, with clinical and fundamental aspects undergoing intensive investigation. Current evidence documents the efficacy and safety of myxoma virus, vesicular stomatitis virus, Newcastle disease virus, Sendai virus, canine adenovirus 2, vaccinia virus, and reovirus in dogs with diverse malignancies in various disease stages. Outcomes of described clinical trials are summarized in Table 2.

Certain heterogeneity exists among the reviewed studies. Some investigations compared dogs with advanced or highly aggressive disease to those with early-stage malignancies. Additionally, several studies lacked distinction between dogs with solid versus hematologic malignancies, despite fundamental differences in pathogenesis and treatment strategies. Furthermore, numerous patients received concurrent medications alongside the designed oncolytic virus, potentially influencing outcomes unpredictably, either enhancing viral antitumor activity or adversely affecting oncolytic properties. These factors require careful consideration when interpreting virotherapy efficacy and safety data.

Notwithstanding these methodological considerations, specific OVs, including NDV, Sendai, Reolysin and VSV, demonstrate substantial potential for treating diverse canine malignancies. The clinical experience accumulated through OV implementation in domestic dogs may prove invaluable for future research, as canines serve as representative models for spontaneous tumor biology [99,100]. Due to substantial similarity between canine and human malignancies [101,102], investigation of viral therapeutic efficacy and systemic effects in dogs, including acute and chronic toxicity, adverse events, and biodistribution, can meaningfully inform human trial design prior to human clinical investigation. Implementing OVs in dogs enables the determination of effective dose ranges, optimal dosing regimens, and ideal administration routes, all crucial parameters for designing efficient human clinical trials.

Oncolytic virotherapy development in veterinary medicine will likely follow the trajectory established in human oncology, albeit with temporal delay. Potential research pathways include the investigation of novel OV delivery systems, evaluation of OV combination with diverse anticancer drug classes, examination of existing OV efficacy and recombination of different OV strains to enhance therapeutic potential.

Enhanced cell-based delivery of OVs to tumors could substantially augment virotherapy efficacy. This approach was previously demonstrated wherein canine mesenchymal stem cells were infected with recombinant adenovirus ICOCAV17 [90], enhancing viral therapeutic efficacy. Diverse cell types, including dendritic cells, neural stem cells, macrophages, and monocytes, can serve as OV delivery systems [103]. Investigations in dogs can provide critical information regarding carrier biodistribution, organ accumulation, and potential adverse effects of this therapeutic strategy. Another promising research direction involves combining viruses with conventional anticancer drugs, potentially generating synergistic cytotoxic effects. Substantial investigation has examined combination therapy with programmed death receptor-1 (PD-1) inhibitors and OVs in vivo [104,105,106].

Investigation of diverse canine viruses for oncolytic potential represents a promising strategy for identifying more effective oncolytic agents. The natural tropism of canine viruses for canine cells represents an inherent advantage for enhanced therapeutic efficacy. Currently, canine morbillivirus and canine distemper virus (CDV) oncolytic activity are being investigated against both human and canine malignancies. The oncolytic activity of vaccine CDV-L strain against canine tubular adenocarcinoma cells has been reported in vitro [107,108]. Investigation of non-pathogenic and vaccine CDV strains in vivo appears warranted, as they represent substantially safer options for clinical application. Crucially, elucidating the impact of pre-existing anti-CDV antibodies on viral-induced oncolysis remains essential.

Compared with other innovative treatments, the introduction of OVs into veterinary medicine is not far behind. Therapeutic agents specifically developed for dogs, such as DNA and RNA vaccines, tumor cell vaccines, CAR-T therapy, cytokine therapy, and others, are at the experimental stage, and only a few have been approved by regulatory authorities.

The list of approved innovative drugs for canine cancer treatment includes a canine plasmid DNA melanoma vaccine Oncept, monoclonal antibodies against CD20, a canine murine CD20 DNA vaccine and T-cell vaccine ELIAS Cancer Immunotherapy.

A murine CD20 DNA vaccine and a canine anti-CD20 mAb [109] are licensed for the treatment of lymphoma, while Oncept is approved for the treatment of oral melanoma [110], and the ELIAS cancer immunotherapy is approved for the treatment of osteosarcoma [111]. Obviously, this list of therapeutics does not cover all needs for treating the wide variety of malignant neoplasms in dogs. Unlike chemotherapeutic drugs, where agents approved for humans can sometimes be used off-label in veterinary medicine, the species specificity of immunotherapy options makes their off-label use essentially impossible, which significantly narrows the range of immunotherapeutic approaches available to patients. In addition, methods such as cell vaccines require highly specialized and expensive manufacturing facilities, making them effective but largely inaccessible treatment options in routine veterinary practice.

The diverse potential applications of OVs, together with their low toxicity, broad therapeutic range, and relative ease of production, make them promising treatment modalities for veterinary oncology.

## 5. Conclusions and Perspectives

Oncolytic virotherapy offers a promising novel approach to cancer immunotherapy in veterinary medicine. While multiple OVs have demonstrated safety and modest efficacy in canine patients, well-designed clinical trials are required to optimize treatment protocols, thoroughly assess therapeutic potential, and expand therapeutic options for canine cancer patients.

## Figures and Tables

**Table 1:** Comparison of oncolytic viruses discussed in this review, including their natural host species, species barriers, replication in canine and human cells, and current applications in humans.

Virus (Family, Genus)	Natural Host Species	Host Range/Species Barrier	Replication in Dogs	Replication in Humans	Clinical Applications in Humans
Myxoma virus (MYXV; *Poxviridae*, *Leporipoxvirus*) [16]	Brazilian cottontail rabbit (*Sylvilagus brasiliensis*)	Naturally restricted to lagomorphs; productive replication is largely confined to rabbit cells, although infection of canine and human tumor cells has been demonstrated under experimental conditions.	Limited/experimental	Limited/experimental	Engineered MYXV constructs are under preclinical and early clinical evaluation as oncolytic agents. [17]
Mammalian orthoreovirus (pelareorep/Reolysin; *Reoviridae*, *Orthoreovirus*) [18]	Rodents; isolates have also been recovered from multiple mammalian species	Broad mammalian host range with no strong species barrier between canine and human hosts.	Yes	Yes	Evaluated in multiple human clinical trials as an oncolytic virus. [19,20]
Vesicular stomatitis virus (VSV-IFNβ-NIS; *Rhabdoviridae*, *Vesiculovirus*) [21]	Livestock, primarily horses, cattle, and swine	Broad experimental host range; natural disease is mainly described in livestock.	Yes	Yes	VSV-based oncolytic constructs, including VSV-IFNβ-NIS, have entered early-phase human clinical trials. [22,23]
Canine adenovirus (CAV; *Adenoviridae*, *Mastadenovirus*) [24,25,26]	Canids	In nature, largely restricted to canids, indicating a marked species barrier to humans.	Yes	No/very limited	No established clinical applications in humans.
Vaccinia virus (VACV; *Poxviridae*, *Orthopoxvirus*) [27]	Not clearly defined; broad mammalian host range	Broad host range with no strict species barrier between dogs and humans under experimental conditions.	Yes	Yes	Used historically as the smallpox vaccine; recombinant VACV strains have also been evaluated as oncolytic agents in human clinical trials. [28,29]
Sendai virus (SeV; *Paramyxoviridae*, *Respirovirus*) [30]	Rodents, especially mice	Natural infection is largely restricted to rodents, but infection of canine and human cells has been demonstrated experimentally.	Limited/experimental	Limited/experimental	Sendai virus-based vectors and constructs have been explored in human clinical studies, including cancer immunotherapy settings. [31,32,33]
Newcastle disease virus (NDV; *Paramyxoviridae*, *Otho-avulavirus* 1) [34]	Avian species	Strong avian host preference; replication in mammalian cells can occur experimentally, but natural infection in mammals is uncommon.	Limited/experimental	Limited/experimental	Several NDV strains have been investigated in human clinical trials as oncolytic agents. [35,36,37,38,39]

**Table 2:** Outcomes of clinical trials of oncolytic viruses in dogs.

Oncolytic Virus	Tumor Types Studied	Administration Route	Observation Period	Clinical Outcome	Adverse Events
Myxoma virus (MYXV) [43]	Soft tissue sarcoma	Intratumoral	28–30 days after treatment	PD	Grade 1/2
Vaccinia virus (TG6002) [51,54]	Solid tumors	Intratumoral	38 days after the treatment	PR, SD	3.3% grade 4 events
Reovirus (Reolysin) [67]	Various types of sarcomas and carcinomas, mastocytoma, Hodgkin’s lymphoma	IV/Intratumoral	Individual	PR	Grade 1/2
VSV-IFNβ-NIS [73,74,76]	Osteosarcoma, lymphomas	Intravenous	28 days after treatment	SD	Grade 1/2 (dose-dependent)
Adenovirus (ICOCAV17) [88,90]	Various types of sarcomas and gliomas, mastocytomas, schwannoma and melanoma	IV	Individual	CR, PR, SD	Grade 1/2
	Gliomas				
Adenovirus (ICOCAV15) [89]	Carcinomas/adenocarcinomas	Intratumoral	1 year after the treatment	PR, SD	Grade 1/2
Sendai virus [95]	Mast cell tumors	Intratumoral	Until the death of the patients	SD	Grade 1/2
NDV [96,97]	Breast cancer	IV	6 days	PR, SD	Grade 1/2
	Cranial tumors	Intratumoral	Until the death of the patients		

IV—intravenous. Clinical outcomes in all studies were evaluated according to RECIST criteria [98]: CR—complete response; PR—partial response; SD—stable disease; PD—progressive disease. Adverse events in all studies were evaluated according to VCOG criteria [75].

## Data Availability

No new data were created or analyzed in this study.
